# Spinal Cord Stimulation Efficacy and Erroneous Conclusions of the Cochrane Library Review of Spinal Cord Stimulation for Low Back Pain by Traeger et al.

**DOI:** 10.3390/brainsci13081181

**Published:** 2023-08-10

**Authors:** Michael Gyorfi, Ian Pillai, Alaa Abd-Elsayed

**Affiliations:** 1 Department of Anesthesiology, University of Wisconsin School of Medicine and Public Health, 600 Highland Avenue, B6/319 CSC, Madison, WI 53792, USA; mgyorfi@uwhealth.org; 2 Midwestern University, 555 31st St, Downers Grove, IL 60515, USA; ipillai@wisc.edu

Neuromodulation, through the use of spinal cord stimulation, is an evolving therapeutic alternative for the management of chronic and refractory pain. Recently, this modality and its efficacy have been called into question when compared to placebo models. Here, we hope to analyze the physiologic fundamentals of the treatment, analyze variables affecting success rate, and highlight unique benefits compared to more standardized practices.

While the exact mechanism of neuromodulation is not entirely understood, the prevailing theory regarding their effectiveness is cited in regard to the “gate control theory of pain.” Taking advantage of the various afferent sensory fibers in the body, the gate control theory seeks to utilize the differing speeds of neural sensation transmission to augment the amount of information conferred between peripheral painful stimuli and the dorsal columns of the central nervous system [1]. Functionally, stimulating larger fibers, A-beta, “closes the gate” on the propagation of painful stimuli to a marked degree and reduces their conscious perception [1]. While still holding theoretical grounds, more recent studies have diverted away, partially, from this theory as the predominate mechanism of spinal cord stimulation effectiveness. It has been repeatedly demonstrated that direct dorsal column stimulation has its own modulatory effects on both sensation and overall perception [2]. It has been demonstrated that both the threshold to tolerance and rate of detection of pain are both increased to a significant degree in patients with spinal cord stimulators [2]. Further studies have even proposed differing mechanisms between ischemic and neurogenic pain, hinting at the differing levels of response exhibited between patient demographics [1]. Although a degree of uncertainty exists as to the mechanism and biochemistry of spinal cord stimulation, this modality nonetheless represents an area of potential for the future of pain management. Further efforts should hope to further elicit aspects that dictate effectiveness between pain type and etiology in hopes of increasing successful patient response. These biochemical models of explanation for spinal cord stimulation are further supported by the current evidence recommendations for intended patient populations, an aspect in and of itself that must be considered prior to intervention.

While spinal cord stimulation has become more accepted for the treatment of chronic and refractory pain, the determination of appropriate candidates remains a challenging limitation. Furthermore, appropriate patient selection has been shown to affect the measured response to spinal cord stimulation, and ultimately whether to pursue continued treatment [3]. As previously mentioned, this etiology is recommended for cases of refractory pain, with failed back surgery syndrome, complex regional pain syndrome, and peripheral vascular disease cases being some of the more classically recommended situations for attempted management [1]. As is expected with nearly any diseased, the demographics affected by these conditions are wide encompassing, and various patient factors can in turn be a limiting aspect of response to intervention. Current analysis has shown a high degree of explantation of devices in patients younger in age, those who were tobacco users, and patients with comorbid psychiatric conditions [4]. Evaluation and analysis, such as this, have helped orient providers on appropriate patient selection and have led to important preoperative managements, such as psychiatric evaluations, becoming commonplace prior to stimulator trials [3]. Similarly, understanding which patient demographics are unlikely to benefit is just as important, with the likes of paraplegic, residual limb pain, and phantom limb pain showing minimal if any reported benefit [1].

By many accounts a minimally invasive procedure, there are adverse effects of stimulator placement. Infection, mechanical failure, repeat surgical placement, among others, are possible outcomes of placement and should be properly discussed with patients [5]. Thankfully, the risk benefit ratio, generally, favors lead placement as most adverse effects are typically minimal in nature and rarely life threatening [5]. Once leads are properly placed and fully functional, additional patient effort is typically required as tuning is often necessary to adjust the stimulation to patient preference to achieve optimal pain relief. Typically, at this point, the majority of the pain that the patient experiences is replaced with a “tingling” sensation or paresthesia [5]. Newer iterations of the technology have thankfully eliminated this aspect as this was a high point of contention for removal for many patient due to discomfort. Regardless of the etiology of pain, an attempted “trial run” of spinal cord stimulation can be attempted, with the option for removal always possible at patient preference; always with the option to pursue more conservative measures if preferred [6]. Reportedly, thirty percent of patients will eventually have their stimulators removed, more specifically ten percent in the first two years [4]. Overall, spinal cord stimulation, and neuromodulation as a whole, represents a strong “middle ground” approach to refractory pain. To add perspective, many patient have tried and failed a host of invasive and conservative measures prior to arrival of stimulator placement; hence, the refractory nature. With failed back surgery syndrome as an example, many patients have already exhausted typical therapeutic and surgical measures, with the major division remaining to continue prolonged therapy likely already started, or attempt a reversible, minimally invasive procedure. This notion can be taken to a further degree within the context of the current opioid epidemic and represents an effective adjunct to chronic opioid use.

Many authors have cited the need for long-term analysis on the effects of stimulation to reasonably determine any sort of effectiveness. Nonetheless, many studies have demonstrated measurable short-term benefits of intervention, especially when compared to alternative practices. Currently, class A evidence has been demonstrated for failed back surgery syndrome, additionally referred to as post-laminectomy syndrome, peripheral neuropathy, angina pectoris, as well as pain due to peripheral ischemia [1]. Classification in this category is based on reviewed analytical models demonstrating a greater than fifty percent reduction in perceived patient pain levels after intervention [7]. Newly emerging retrospective studies have continued to echo this clinical significance and efficacy for these assigned conditions, while at the same time showing potential limiting areas of use, such as generic low back pain [8]. While a host of class A evidence is present for a variety of conditions, lesser degrees exist, such as class B for complex regional pain syndrome, and mark points of interest in need of further development [1]. Currently, the highest degree of efficacy for the use of spinal cord stimulation exists for short-term iteration; often described within a six-month window from implantation [3]. As such, long-term implications and the degree of effectiveness remains a high point of contention among academics and providers. Regardless, new and emerging studies have attempted to argue for a prolonged effect of benefit, including improvements in patient experience outside of the general pain level [9]. General improvements in patient quality of life, accessory or maintenance medication use, and minimal adverse effects of stimulator placement have been reported in refractory patients [9].

The full context and breadth of the available literature must be taken into account when making general recommendations about the use of SCS in the real world. Clinical trials contrasting SCS with a placebo or “no treatment” (including studies using conventional medical management (CMM) with a parallel-group design) were included in the search criteria created by the authors. Large, multicenter comparative effectiveness trials and pragmatic studies, such as those contrasting SCS with revision decompression and/or fusion surgery and tonic SCS with novel waveforms, were however excluded.

We agree that placebo- and sham-controlled trials provide the strongest available scientific evidence, but paresthesia-free waveforms that allow for randomized, double-blind studies were only created approximately ten years ago. The cost of conducting these studies and the difficulty in recruiting participants for sham-controlled SCS trials have made it difficult for independent physician investigators to finish them. The US Food and Drug Administration does not require such studies for regulatory approval in the presence of a predicate device. Such studies are expensive and difficult to enroll patients in, and the risk–benefit ratio does not favor businesses and their shareholders, discouraging the industry from carrying out such studies. The lack of published research in this area and the small, single-center design of the few published sham-controlled studies can both be attributed to these historical constraints.

Traeger et al. identified parallel trials comparing SCS and conventional medical management (CMM) to CMM alone, but their analysis of these studies and handling of inclusion/exclusion were flawed. Kumar et al. Rigoard et al., and Kapural et al., were three parallel trials with medium-term follow-up that were initially included in a sub-analysis [10,11,12].

In this case, the authors compared different treatment frequencies. Both Kumar et al. and Rigoard et al. used older, conventional stimulation waveforms that are mechanistically distinct and less efficient than the high-frequency (10 kHz) stimulation used in Kapural et al. They combined outcome data pertinent to various body regions in addition to grouping different treatments. With the primary goal of achieving a 50% reduction in leg pain, Kumar et al. included patients in their study who had predominant leg pain in relation to CLBP (importantly, the predominance of CLBP was a major reason for exclusion) [10,11,12].

In spite of this, Traeger et al. performed a combined analysis of the three studies. Compared to participants who only received CMM, participants who received SCS were 7.4 times more likely to report a 50% or greater improvement in pain. The estimated risk ratio was then reduced to 4.2 by the authors by excluding the Kapural et al. study from the secondary analysis. They used I2 statistical analysis, which is appropriate when analyzing many meta-analyses but inappropriate when analyzing only three studies, to support their claim that there was heterogeneity and an excessively large effect size. Furthermore, only Rigoard et al. and Kapural et al. would have remained in the sub-analysis if the Kumar et al. study had been eliminated at the outset as a study of leg pain. Eliminating an outlier when there are only two studies raises the concern that Traeger et al. is hand-selecting studies to support their bias [8,10,11,12]. 

As is the case with many topics in medicine, chronic pain is a topic of increasing complexity. Today, pain management is a true multimodal approach, often with personalized treatment regimens to best serve the patient at hand. Here, we highlight spinal cord stimulation as a possible supplement, if not adjunct, to chronic refractory pain. With the efficacy established here, spinal cord stimulation, along with continued medical management, is a modality best considered for those who have been markedly debilitated by their refractory pain. Moving forward, further efforts should be made to optimize the technological aspect of the practice and hopefully elicit the potential benefit to new emerging high frequency vs. burst patterns of stimulation [13]. 

It is difficult to elude the true value of neuromodulation with our current body of research despite having proper patient selection. To further elicit a more accurate value of neuromodulation, objective values such as molecular, digital, electrophysiology, neuroimaging, and autonomic testing need to be further studied. Efforts should be made to understand the decay in the perceived and reported effectiveness with the time of the implanted stimulation. Lastly, cost continues to remain a constant limiting factor despite the fact that prolonged conservative management is often more costly than stimulator intervention [3]. With optimism, the future of spinal cord stimulation should hope to address some of these limitations and remain a viable option for those in pain.

## References

[1] Garcia K., Wray J.K., Kumar S. (2023). Spinal Cord Stimulation. [Updated 2022 July 10]. StatPearls.

[2] Sdrulla A.D., Guan Y., Raja S.N. (2018). Spinal Cord Stimulation: Clinical Efficacy and Potential Mechanisms. Pain Pract..

[3] Rock A.K., Truong H., Park Y.L., Pilitsis J.G. (2019). Spinal Cord Stimulation. Neurosurg. Clin. N. Am..

[4] Dougherty M.C., Woodroffe R.W., Wilson S., Gillies G.T., Howard M.A., Carnahan R.M. (2021). Risk Factors and Survival Analysis of Spinal Cord Stimulator Explantation. Neuromodulation.

[5] Fontaine D. (2021). Spinal cord stimulation for neuropathic pain. Rev. Neurol..

[6] Malige A., Sokunbi G. (2019). Spinal Cord Stimulators: A Comparison of the Trial Period Versus Permanent Outcomes. Spine.

[7] Higashiyama N., Tamura S., Sugawara T. (2023). Efficacy of Spinal Cord Stimulation for Failed Back Surgery Syndrome in Elderly Patients: A Retrospective Study. Pain Res. Manag..

[8] Traeger A.C., Gilbert S.E., Harris I.A., Maher C.G. (2023). Spinal cord stimulation for low back pain. Cochrane Database Syst. Rev..

[9] Vervaat F.E., VanDerGaag A., Teeuwen K., Van Suijlekom H., Dekker L., Wijnbergen I.F. (2023). Long-term efficacy and safety of spinal cord stimulation in patients with refractory angina pectoris. Int. J. Cardiol. Heart Vasc..

[10] Kumar K., Taylor R.S., Jacques L., Eldabe S., Meglio M., Molet J., Thomson S., O’callaghan J., Eisenberg E., Milbouw G. (2007). Spinal cord stimulation versus conventional medical management for neuropathic pain: A multicentre randomised controlled trial in patients with failed back surgery syndrome. Pain.

[11] Rigoard P., Basu S., Desai M., Taylor R., Annemans L., Tan Y., Johnson M.J., Abeele C.V.D., North R. (2019). PROMISE Study Group; Multicolumn spinal cord stimulation for predominant back pain in failed back surgery syndrome patients: A multicenter randomized controlled trial. Pain.

[12] Kapural L., Jameson J., Johnson C., Kloster D., Calodney A., Kosek P., Pilitsis J., Bendel M., Petersen E., Wu C. (2022). Treatment of nonsurgical refractory back pain with high-frequency spinal cord stimulation at 10 kHz: 12-month results of a pragmatic, multicenter, randomized controlled trial. J. Neurosurg. Spine.

[13] London D., Mogilner A. (2022). Spinal Cord Stimulation: New Waveforms and Technology. Neurosurg. Clin. N. Am..

